# Circ_SMAD4 promotes gastric carcinogenesis by activating wnt/β‐catenin pathway

**DOI:** 10.1111/cpr.12981

**Published:** 2021-01-17

**Authors:** Liyan Wang, Bin Li, Xiaoyuan Yi, Xuhua Xiao, Qinghua Zheng, Lei Ma

**Affiliations:** ^1^ Digestive Department Affiliated Hospital of Guilin Medical College Guilin City Guangxi Zhuang Autonomous Region China

**Keywords:** circ_SMAD4, CTNNB1, gastric cancer, miR‐1276, TCF4, Wnt, β‐catenin pathway

## Abstract

**Objectives:**

Circular RNAs (circRNAs) are essential participants in tumour progression. This study focused on investigating the mechanism of a novel functional circRNA in gastric cancer (GC).

**Methods:**

Gene expression was detected by qRT‐PCR or Western blot. Survival curves were generated via Kaplan‐Meier method. In vitro and in vivo assays were used to investigate the impact of circ_SMAD4 on GC cell growth and tumorigenesis. Agarose gel electrophoresis assay, RNase R treatment and Sanger sequencing were utilized for confirming the circular structure of circ_SMAD4. Relationship between molecules was monitored by a series of mechanical experiments, as needed.

**Results:**

Circ_SMAD4 expression was potentiated in GC. Circ_SMAD4 depletion impeded GC cell growth in vitro and restrained tumorigenesis in vivo. Mechanically, nuclear circ_SMAD4 recruited TCF4 to facilitate CTNNB1 transcription, while cytoplasmic circ_SMAD4 sequestered miR‐1276 to prevent the silence of CTNNB1 mRNA, leading to activation of Wnt/β‐catenin pathway. Rescue experiments validated that circ_SMAD4 depended on miR‐1276/TCF4‐regulated CTNNB1 to elicit accelerating effects on GC cell growth.

**Conclusion:**

Circ_SMAD4 facilitated GC tumorigenesis by activating CTNNB1‐dependent Wnt/β‐catenin pathway. Hopefully, the findings could provide new clues for improving GC prognosis and treatment.

## Graphical abstract

Circ_SMAD4 sequesters miR‐1276 and recruits TCF4 to promote CTNNB1 expression to activate WNT/β‐catenin signalling, therefore accelerating gastric carcinogenesis.

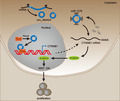

## 1 INTRODUCTION

Gastric cancer (GC), severely prevalent in East Asian regions, raises societal healthy concerns.^1^, ^2^ GLOBOCAN database has illustrated the high incidence and mortality of GC.^3^, ^4^, ^5^ Besides, the survival of GC patients in 5 years is generally dreadful due to the lack of early typical symptoms, albeit with progresses in treatments.^6^ It is difficult to improve the efficacy of GC treatment owing to intricate genetic and epigenetic alterations. Therefore, grasping the molecular mechanisms behind GC development is highly urgent for GC treatment.

Circular RNAs (circRNAs) are formed upon special and selective shearing. Unlike linear RNAs, circRNAs basically originate from the exons or introns of their parental genes.^7^, ^8^ Typically, the abnormal expression of circRNAs is associated with tumour aggravation. Wang et al stated that circ_0091570 inhibits hepatocellular cancer cell growth.^09^ Meanwhile, versatile physiological regulations of circRNAs on protein‐coding genes are introduced as they can interact with certain RNAs and proteins.^10^ Among these regulation modes, circRNAs acting as miRNA sponges for the protection of mRNA translation are well‐reported in cancers including GC.^10^, ^11^, ^12^ For instance, circ_006100 elevates GPRC5A expression via sponging miR‐195, resulting in GC aggravation.^13^, ^14^ Another common mechanism behind circRNA regulating gene expression involves RNA binding proteins (RBPs).^15^, ^16^ As an example, nuclear circRNA_102171 interacts with CTNNBIP1 to activate WNT/β‐catenin pathway in papillary thyroid cancer.^17^ However, preliminary documents still lack thorough explanation of most circRNAs in GC.

As a classic pathway, Wnt/β‐catenin signalling is frequently activated in the tumorigenesis of diverse cancers, including GC.^18^ For instance, LINC01133 inactivates Wnt/β‐catenin pathway to impair GC progression via miR‐106a‐3p/APC axis.^19^ As is well‐known, the hallmark of Wnt/β‐catenin activation is the nuclear accumulation of β‐catenin.^20^ However, whether circRNAs mediate modulation on this pathway in GC still requires deeper understanding.

With the employment of microarray analysis, circ_SMAD4 was unveiled to be overexpressed in GC. Therefore, present study aimed at unravelling the function and mechanism of circ_SMAD4 in GC tumorigenesis. The present study might provide the potential for circ_SMAD4 to be a technically effective biomarker for GC therapies.

## 2 MATERIALS AND METHODS

### 2.1 Microarray

CircRNA microarray analysis was undertaken with Human CircRNA Array v2.1 (CapitalBio, Beijing, China). The differentially expressed circRNAs were deemed to be significantly different between groups in line with following conditions: fold change > 2.0 and *P* < 0.05.

### 2.2 Tissue samples

40 pairs samples of GC tissues and adjacent non‐tumour tissues were attained from patients at Affiliated Hospital of Guilin Medical College. Right after surgical excision, tissue samples were processed by liquid nitrogen, followed by storage at −80℃. Experimental procedures were licensed by the Ethics Committee of Affiliated Hospital of Guilin Medical College. Written informed consents were attained from all patients enrolled in. Before excision, no patients received chemotherapy or radiotherapy.

### 2.3 Cell culture

Gastric epithelial cell line (GES‐1) and human GC cell lines (SGC‐7901, MKN45, MGC‐803, AGS and BGC‐823) were purchased from American Type Culture Collection (ATCC; Manassas, VA, USA). Cells were maintained at 37℃ with 5% CO_2_ in RPMI‐1640 (GE Healthcare Life Sciences, Logan, UT, USA) supplying 1% penicillin/streptomycin (Solarbio, Beijing, China) and 10% FBS (Gibco, Rockville, MD, USA) in a humidified incubator. To activate Wnt/β‐catenin pathway, AGS or BGC‐823 cells were treated for 24 hours with 20 mM lithium chloride (LiCl; Sigma‐Aldrich, St. Louis, MO, USA).

### 2.4 Cell transfection

Specific shRNAs against circ_SMAD4 (sh/circ_SMAD4#1/2) or TCF4 (sh/TCF4#1/2) and their corresponding negative control (NC) (sh/ctrl) were constructed by Genechem (Shanghai, China). In addition, the pcDNA3.1/TCF4 or pcDNA3.1/CTNNB1 was obtained through subcloning indicated cDNA into pcDNA3.1 vectors (Invitrogen), while pcDNA3.1 Mini (+) vector (Addgene) was applied for circ_SMAD4 overexpression, with the corresponding empty vector as NCs. Moreover, miR‐1276 mimics, miR‐1276 inhibitors, NC mimics and NC inhibitors were constructed by GenePharma (Shanghai, China). AGS or BGC‐823 cells were individually transfected with these plasmids through Lipotransfectamine 3000 (Thermo Fisher Scientific, Waltham, MA, USA). The sequences of shRNAs against circ_SMAD4 were as follows:

sh‐ctrl: 5′‐CCGCGCCAACTAAGCGGTACAATGCTCGAGCATTGTACCGCTTAGTTGGCTTTTTG‐3′;

sh‐circ_SMAD4#1:5′‐CCGCGCCAACTAAGTCTGTCAGCTGCTCGAGCAGCTGACAGACTTAGTTGGCTTTTTG‐3′;

sh‐circ_SMAD4#2:5′‐CCGCGAAAGCCAACTAAGTCTGTCACTCGAGTGACAGACTTAGTTGGCTTTCTTTTTG‐3′.

### 2.5 qRT‐PCR

Total RNA was isolated by TRIzol reagent (Invitrogen, Carlsbad, CA, USA) and reversely transcribed adopting GoScript Reverse Transcription System (Qiagen GmbH, Germany). qRT‐PCR was implemented on ABI 7900 Detection System (Applied Biosystems, Foster City, CA, USA) using a SYBR‐Green PCR Master Mix kit (Takara, Dalian, China). Relative gene expression normalized to U6 or GAPDH was determined via 2^−ΔΔCt^ approach. Primer sequences used were as follows:

circ_SMAD4 (Convergent): 5′‐GGGACCGGATTACCCAAG‐3′ (forward), 5′‐GTTAAGGGCCCCAACGG‐3′ (reverse); circ_SMAD4 (Divergent): 5′‐TGCCTGGTTAAAGTCTGTGG‐3′ (forward), 5′‐GCATAAGCGACGAAGGTCAT‐3′ (reverse).

### 2.6 Actinomycin D and RNase R treatments

For RNase R treatment, total RNA was cultivated without or with RNase R (Epicentre, Madison, WI, USA) for 30 minutes at 37℃. The relative levels of circ_SMAD4, linear SMAD4 and GAPDH were assayed by qRT‐PCR, normalizing to those measured in mock group. To measure mRNA stability, cells were subjected to 2 mg/ml of Actinomycin D (Sigma‐Aldrich) or dimethylsulphoxide (DMSO; negative control). Total cellular RNA was extracted at specific times (0, 2, 4, 8, 12 hours). Results were analysed by 2^−ΔΔCt^ approach. Ct values from triplicate samples were normalized to the internal housekeeping transcript and were then normalized to that of samples before adding Actinomycin D. Results were shown as the ratio of mRNA abundance at indicated times relative to 0 hour.

### 2.7 Colony formation assay

Colony formation assay was implemented to assess the proliferation of indicated AGS or BGC‐823 cells. Cells (800 per well) were added in 6‐well plates and cultured for 14 days. Afterwards, colonies were fixed in methanol (Sigma‐Aldrich), stained by crystal violet (Sigma‐Aldrich) and calculated manually.

### 2.8 EdU assay

4 × 10^4^AGS or BGC‐823 cells were incubated for 2 hours with 50 μm EdU (RiboBio, Nanjing, China) and then treated with Apollo (RiboBio) and DAPI (Sigma‐Aldrich). The proportion of EdU‐positive cells was assessed under a fluorescence microscopy (Nikon, Tokyo, Japan).

### 2.9 Flow cytometry analysis

Cells in 6‐well plates were washed twice in cold PBS (Sigma‐Aldrich) and re‐suspended in 1 × binding buffer (Invitrogen). Cells were sequentially incubated for 15 minutes with Annexin V‐FITC/PI (Invitrogen) away from light and finally analysed applying the Accuri C6 flow cytometer (BD Biosciences, Franklin Lakes, NJ, USA).

### 2.10 Xenograft model

Six‐week‐old BALB/c nude mice were obtained from Shi Laike Company (Shanghi, China). GC cells transfected with sh/circ_SMAD4#1 or sh/ctrl were injected into mice, subcutaneously. Tumour volume was recorded every 4 days. Mice were killed and dissected at 4 weeks following injection, and the tumours were weighed. Animal assay was conducted with the approval of the Animal Ethics Committee of Affiliated Hospital of Guilin Medical College.

### 2.11 Immunohistochemistry (IHC)

Tissues gained from xenograft model were put in formalin (Sigma‐Aldrich), fixed and paraffin‐embedded. Following deparaffinating and rehydrating, sections were incubated with primary antibodies against Ki67 (Abcam, Cambridge, USA) or PCNA (Abcam) and corresponding secondary antibodies. Following staining using DAB (Sigma‐Aldrich), sections were observed under a light microscopy (Nikon).

### 2.12 Western blot

Following previous description, Western blot was conducted.^21^ Primary antibodies against β‐catenin (ab16051, Abcam), c‐myc (ab39688, Abcam), CCND1 (ab226977, Abcam), SMAD4, TCF4, Histone 3 and GAPDH (ab8245, Abcam) were employed. Secondary antibody was anti‐rabbit IgG conjugated with HRP (ab205718, Abcam).

### 2.13 Subcellular fractionation

According to the previous protocol,^22^ RNA in the cytoplasm and nucleus of GC cells was isolated with a PARIS™ Kit (Ambion, Austin, TX, USA). Isolated RNA was assayed utilizing qRT‐PCR, with GAPDH or U6 as the cytoplasmic or nuclear control, respectively.

### 2.14 Fluorescence in situ hybridization (FISH)

FISH assay was implemented in line with the procedures described previously.^23^ Alexa Fluor 555‐labeled circ_SMAD4 probe was synthesized by RiboBio. A Fluorescent in Situ Hybridization Kit (RiboBio) was attained to study the probe signals. Images were examined applying the fluorescence microscopy.

### 2.15 Luciferase reporter assay

The pGL3‐CTNNB1 promoter vector (Promega, Madison, WI, USA) was co‐transfected into cells with indicated transfection plasmids. The pmirGLO dual‐luciferase vectors (Promega) containing circ_SMAD4 or CTNNB1 3′UTR sequence were designed and co‐transfected into cells with indicated miRNA mimics or NC mimics. In addition, pmirGLO‐CTNNB1‐WT/MUT or pmirGLO‐circ_SMAD4‐WT/MUT vectors were constructed using the full‐length CTNNB1 3′‐UTR or circ_SMAD4 sequence with wild‐type (WT) or mutant (MUT) miR‐1276 binding sites, followed by co‐transfection into cells with miR‐1276 mimics or NC mimics. Dual‐luciferase assay system (Promega) was utilized for examining the luciferase activity 48 hours post‐co‐transfection.

### 2.16 RNA Pull‐down assay

RNA pull‐down assay was implemented with the Pierce™ Magnetic RNA‐protein pull‐down kit (Thermo Fisher Scientific). The pull‐down probe of circ_SMAD4 was the oligonucleotide containing complementary binding site for the spliced junction region of circ_SMAD4. Biotin‐labelled RNA was mixed with cell extracts, with the mixture containing non‐biotin RNA as the NC. The mixtures were incubated with Dynabeads Myone Streptavidin T1 beads. Then, proteins in the RNA‐protein complex were eluted and separated by SDS‐PAGE (Bio‐Rad, Hercules, CA, USA). Afterwards, the gel was stained using a Fast Silver Stain Kit (Beyotime, Shanghai, China), followed by mass spectrometry.

### 2.17 RNA Immunoprecipitation (RIP)

RIP was undertaken using a Magna RIP Kit (Millipore, Billerica, MA, USA). AGS or BGC‐823 cells were lysed utilizing RIP lysis buffer. For Ago2‐RIP, cell extract was incubated in RIP buffer adding magnetic beads (Invitrogen) conjugated with anti‐Ago2 (Millipore) or anti‐IgG (Millipore). For detecting the interaction of circ_SMAD4 with TCF‐4, cell lysate was cultured with anti‐TCF‐4 (Millipore), anti‐SNRNP70 (positive control; Millipore) or anti‐IgG (negative control). qRT‐PCR was applied for analysing RNAs in immunoprecipitates.

### 2.18 Chromatin immunoprecipitation (ChIP)

As described previously, ChIP experiments were executed.^24^ Cross‐linked chromatin was initially sonicated to fragments of 500‐bp. Immunoprecipitation was conducted adopting antibodies against TCF4 or IgG (negative control). Following adding magnetic beads, the precipitated chromatin fragments were purified, isolated and examined via qRT‐PCR.

### 2.19 Electrophoretic mobility shift assay (EMSA)

EMSA experiment was conducted as depicted previously.^25^ The nuclear extracts were obtained from AGS cells. Probes were produced by annealing single‐strand oligonucleotides containing the TCF4 consensus sequence of circ_SMAD4 promoter and labelling the ends with [γ‐32P] ATP using T4 polynucleotide kinase (TaKaRa Bio). Anti‐TCF4 (Proteintech) and anti‐IgG (Santa Cruz) were used as primary antibodies.

### 2.20 Statistical analysis

Results shown as mean ± SD were derived from at least three independent assays and imported into SPSS 22.0 (IBM, Chicago, IL, USA). The Kaplan‐Meier and log‐rank rest method was applied for survival analysis. Correlations between RNAs were analysed via Pearson's correlation analysis. Differences in experimental variables were confirmed via Student's *t* test or one‐way ANOVA, with the significant level of *P* < 0.05.

## 3 RESULTS

### 3.1 Characterization of circ_SMAD4 level and its stable structure

**FIGURE 1:**
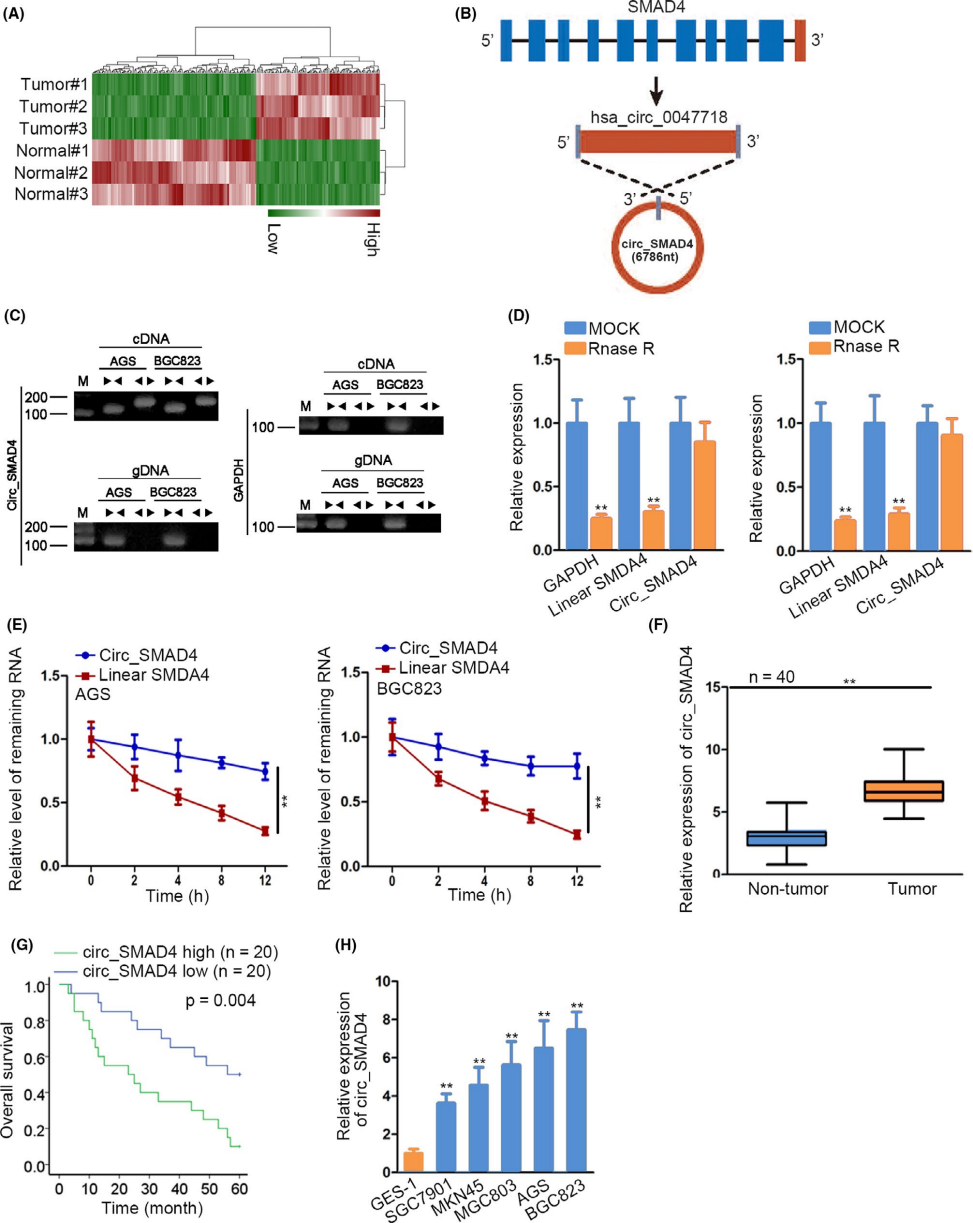
Characterization of circ_SMAD4 level and its stable structure. A, In three pairs GC tissues versus controls, the dysregulated circRNAs from microarray analysis were presented as heat map. B, Head‐to‐tail splicing and the origin gene of circ_SMAD4 were schematically illustrated and confirmed. (C‐E) AGE analysis and RNase R/actinomycin D treatments tested the circular form of circ_SMAD4. F, The expression of circ_SMAD4 was detected by qRT‐PCR in 40 GC tissues versus non‐tumorous samples. G, Kaplan‐Meier curve depicted the survival of GC patients with high or low level of circ_SMAD4. H, qRT‐PCR monitored circ_SMAD4 expression in GC cell lines and non‐cancerous GES‐1 cells. **P < 0.01

To search dysregulated circRNAs in GC, microarray analysis was carried out in 3 GC tissues and paired non‐tumour samples. Consequently, 86 circRNAs exhibited elevated levels and 114 exhibited suppressed levels in GC tissues relative to controls. We focused on the most overexpressed circRNA and hsa_circ_0047718 (Figure 1A). Hsa_circ_0047718 was derived from SMAD4 (called circ_SMAD4 subsequently) and had 6786 nt in length (Figure 1B), and its splice junction forming the circular structure was determined by Sanger sequencing (Supporting Information Figure S1A). Agarose gel electrophoresis (AGE) assay displayed that circ_SMAD4 was amplified only from cDNA with divergent primers but could not be found in the products from genomic DNA (Figure 1C and Supporting Information Figure S1B‐C). In addition, it was proved that circ_SMAD4 was more stable than linear SMAD4 (Figure 1D‐E). Thereafter, qRT‐PCR data depicted a higher circ_SMAD4 expression in 40 GC tissues in contrast to matched non‐cancerous samples (Figure 1F), so was the level of linear SMAD4 (Supporting Information Figure S1D). More importantly, high circ_SMAD4 level predicted dreadful survival in GC patients (Figure 1G). Consistently, circ_SMAD4 level was also increased in GC cells compared with normal GES‐1 cells (Figure 1H). To summarize, circ_SMAD4 was upregulated in GC.

### 3.2 Loss of circ_SMAD4 blunted GC tumorigenesis

**FIGURE 2:**
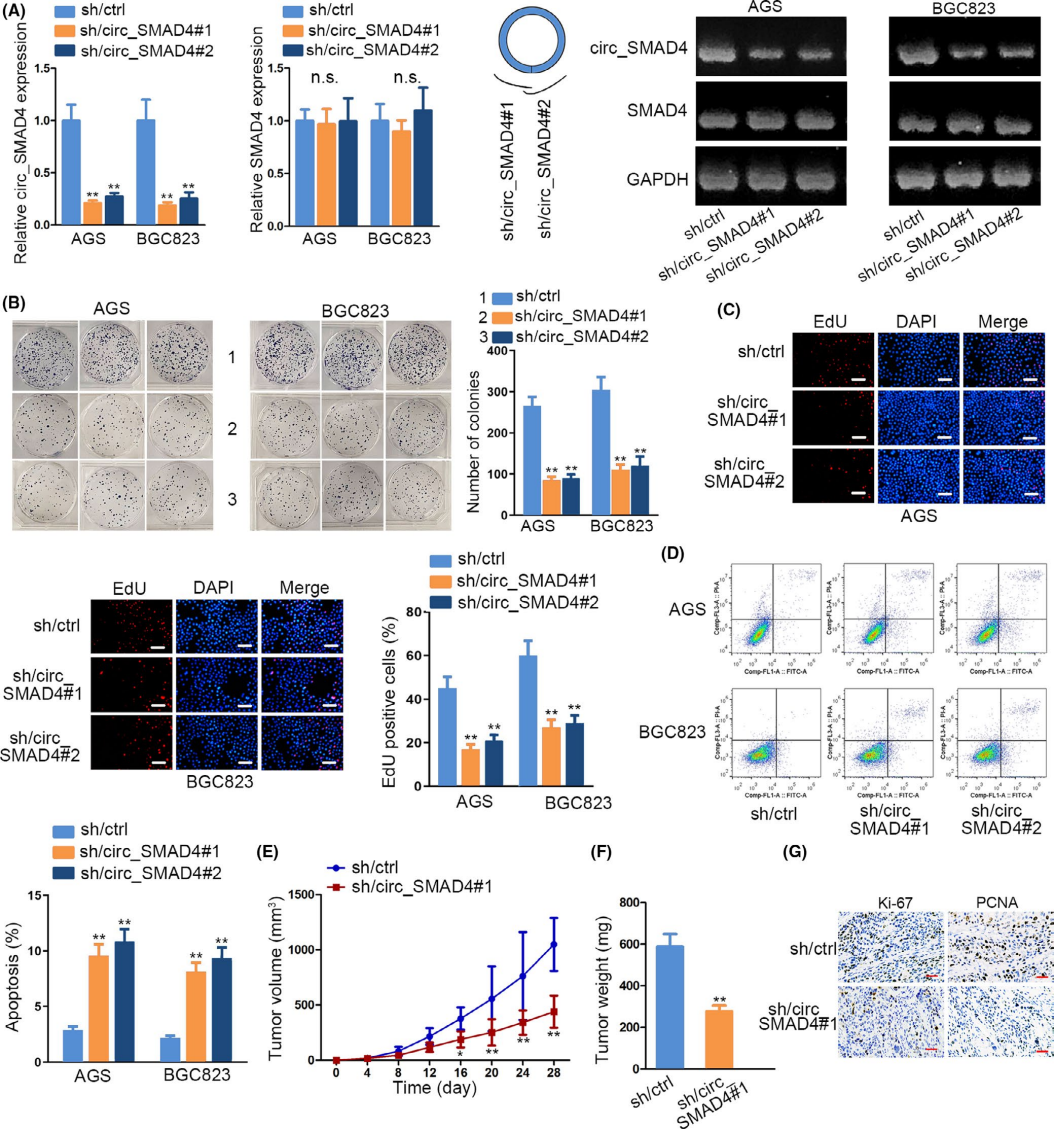
Loss of circ_SMAD4 blunted GC tumorigenesis. A, Circ_SMAD4 or SMAD4 expression in AGS and BGC823 cells was detected by qRT‐PCR and AGE after transfection with sh/circ_SMAD4#1/2. B, Images and quantification of colonies formed under circ_SMAD4 interference. C, Images and quantification of EdU‐stained AGS and BGC823 cells under circ_SMAD4 deficiency. D, The influence of circ_SMAD4 depletion on AGS and BGC823 cell apoptosis was assessed via flow cytometry analysis. (E‐F) The growth curve and weight of subcutaneous xenograft tumours obtained from nude mice under sh/ctrl and sh/circ#1 sets. G, IHC staining evaluated Ki‐67 and PCNA expressions in tumours with or without circ_SMAD4 silencing. *P < 0.05, **P < 0.01. n.s. indicated no significance

In vitro and in vivo assays were executed to probe the role of circ_SMAD4 in GC. First, we confirmed that circ_SMAD4 not SMAD4 was interfered in AGS and BGC823 cells by specific shRNAs targeting the splice junction of circ_SMAD4 (Figure 2A and Supporting Information Figure S2A). Interestingly, silencing circ_SMAD4 caused impediment to GC cell proliferation and induction of GC cell apoptosis (Figure 2B‐D). By contrast, overexpressing circ_SMAD4 in SGC7901 and MKN45 cells strengthened the proliferative abilities of both cells (Supporting Information Figure S2B‐D). Later, in vivo xenograft experiments were also implemented. As anticipated, the growth rate of tumours from circ_SMAD4‐silenced GC cells was much slower and the tumour weight was lighter than tumours formed in control group (Figure 2E‐F). In addition, the positivity of two proliferative markers, Ki‐67 and PCNA, was also lessened in tumours with circ_SMAD4 deficiency (Figure 2G). In summary, depletion of circ_SMAD4 blunted GC tumorigenesis.

### 3.3 Circ_SMAD4 activated WNT/β‐catenin pathway

**FIGURE 3:**
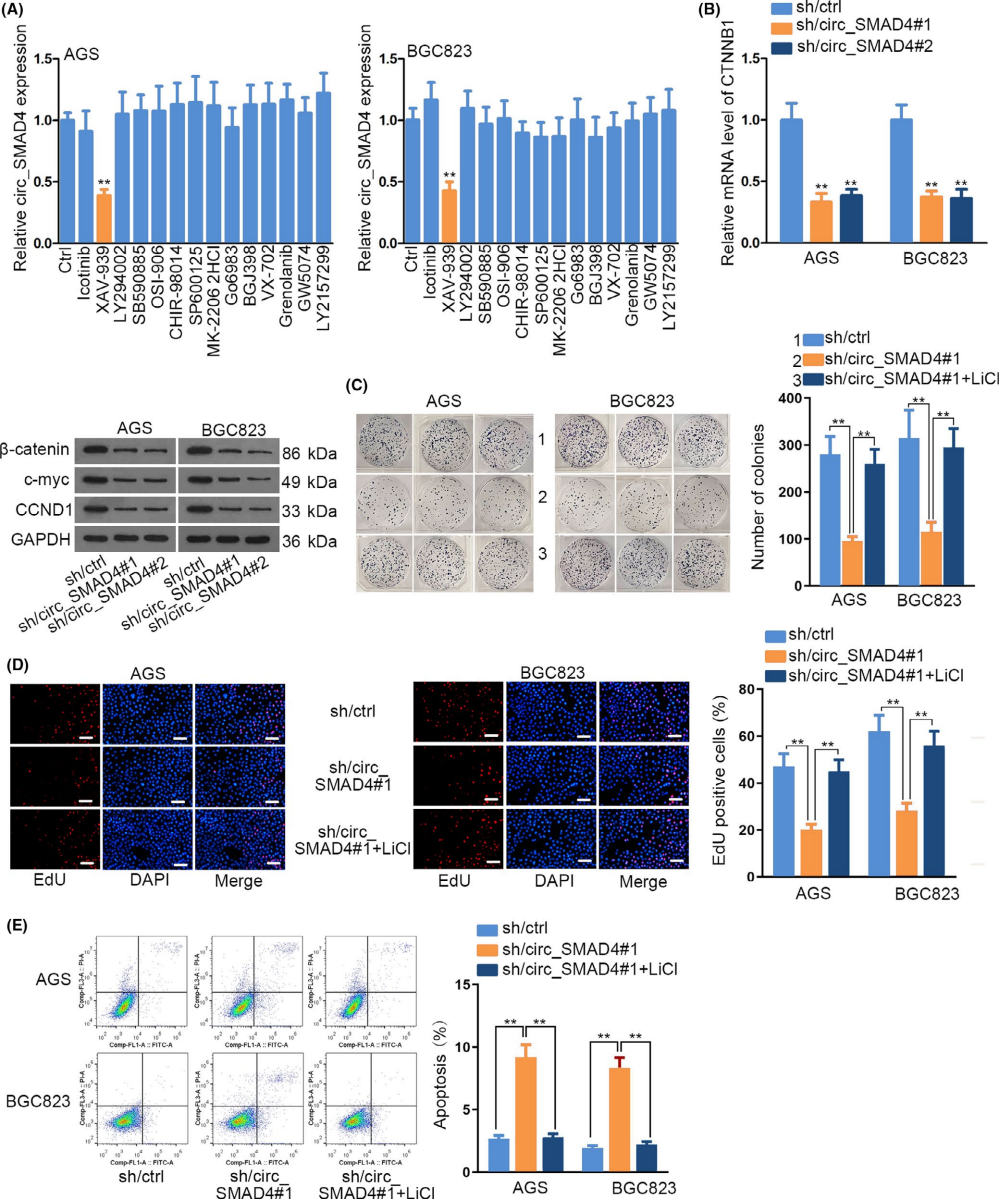
Circ_SMAD4 affected the activation of WNT/β‐catenin pathway. A, Circ_SMAD4 expression was investigated upon the application of several pathway inhibitors in AGS and BGC823 cells. B, CTNNB1 mRNA and protein (β‐catenin), and WNT/β‐catenin pathway‐related proteins (CCND1 and c‐myc) were determined after circ_SMAD4 blockade by using qRT‐PCR or Western blot. (C‐D) Colony formation and EdU assays proved that LiCl treatment rescued the hindered AGS and BGC823 cell proliferation caused by circ_SMAD4 inhibition. E, Flow cytometry analysed that LiCl abrogated the effects of circ_SMAD4 blockade on AGS and BGC823 cell apoptosis. **P < 0.01

CircRNAs have been increasingly documented to modulate functional pathways to affect tumour growth.^13^, ^26^, ^27^ Intriguingly, an obvious reduction of circ_SMAD4 expression was only observed in AGS and BGC‐823 cells upon the treatment of XAV‐939, the inhibitor of WNT/β‐catenin pathway (Figure 3A). Moreover, XAV‐939 treatment weakened cell proliferation while stimulated cell apoptosis (Supporting Information Figure S3A‐C). Of note, such phenomena induced by XAV‐939 were cancelled in response to overexpressed circ_SMAD4 (Supporting Information Figure S4A‐C). Next, to probe whether circ_SMAD4 could regulate WNT/β‐catenin pathway in GC, we investigated the potential regulation of circ_SMAD4 on CTNNB1. Unsurprisingly, CTNNB1 expression was significantly higher in GC tissues (Supporting Information Figure S5A). Importantly, CTNNB1 level was positively correlated with circ_SMAD4 in these 40 GC samples (Supporting Information Figure S5B). Moreover, circ_SMAD4 deficiency impaired CTNNB1 level and also decreased the levels of β‐catenin, CCND1 and c‐myc proteins (Figure 3B and Supporting Information S5C). Then, we conducted rescue experiments via WNT/β‐catenin agonist, LiCl. Results demonstrated that the effects of circ_SMAD4 interference on GC cell proliferation and apoptosis were counteracted after LiCl treatment (Figure 3C‐E). Altogether, circ_SMAD4 activated WNT/β‐catenin pathway to expedite GC cell growth.

### 3.4 circ_SMAD4 bound to TCF‐4 in GC cells

**FIGURE 4:**
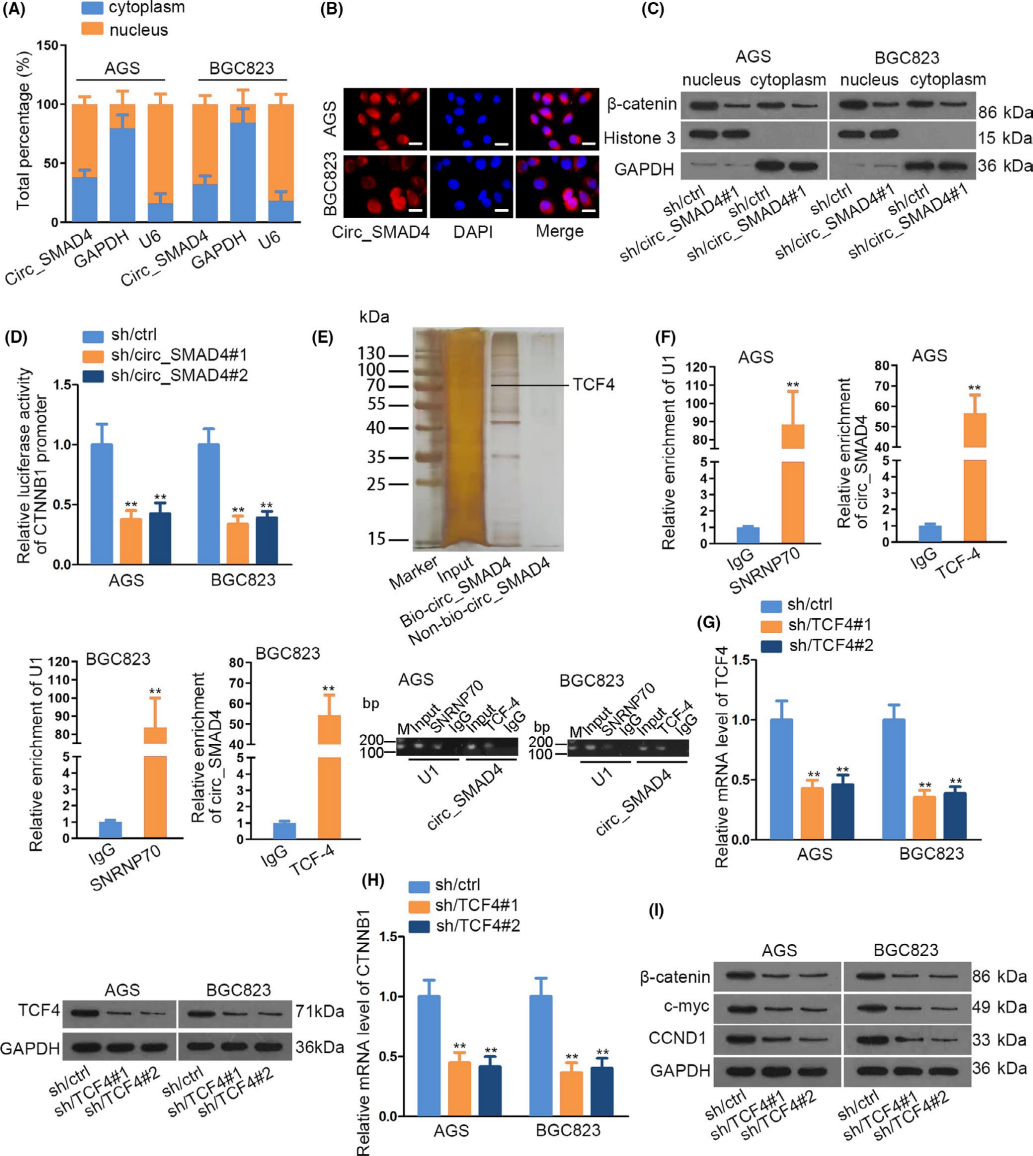
Circ_SMAD4 bound to TCF‐4. (A‐B) The distribution of circ_SMAD4 in AGS and BGC823 cells was assayed through subcellular fractionation plus qRT‐PCR and immunofluorescence staining. C, Nuclear and cytoplasmic β‐catenin level in two GC cells was assessed by Western blot. D, Under the absence of circ_SMAD4, CTNNB1 promoter activity was assayed by means of luciferase reporter assay. E, RNA pull‐down and mass spectrometry interrogated circ_SMAD4‐binding proteins. F, RIP and AGE confirmed that circ_SMAD4 bound to TCF4 protein. SNRNP70 and U1 as positive controls. G, TCF4 was effectively knocked down as illustrated via qRT‐PCR and Western blot. (H‐I) The impacts of TCF4 downregulation on regulating CTNNB1, c‐myc, CCND1 and β‐catenin levels were appropriately validated through qRT‐PCR and Western blot. **P < 0.01

Since subcellular location of circRNAs can be informative to their regulatory mechanism, we subsequently examined circ_SMAD4 distribution in GC cells. It was uncovered that circ_SMAD4 existed in both the nuclear and cytoplasmic fractions of GC cells, although with a larger proportion in nucleus (Figure 4A‐B). It was depicted that inhibition of circ_SMAD4 reduced the level of both cytoplasmic and nuclear β‐catenin in AGS and BGC823 cells (Figure 4C). Such data implied that circ_SMAD4 might regulate total β‐catenin expression but not directly affect the transport of β‐catenin, which echoed the previous observation that CTNNB1 level was restrained by circ_SMAD4 knock‐down. Therefore, we then probed into how circ_SMAD4 influenced CTNNB1 expression in GC. Results suggested that circ_SMAD4 depletion evidently reduced the luciferase activity of CTNNB1 promoter (Figure 4D), indicating a possible transcriptional regulation of circ_SMAD4 on CTNNB1. Hence, the circ_SMAD4‐binding proteins were surveyed via RNA pull‐down and mass spectrometry. It turned out that transcription factor 4 (TCF4) was abundant in Bio‐circ_SMAD4 group (Figure 4E). Next, the interaction of circ_SMAD4 with TCF4 was further certified by RIP assays, RNA pull‐down and EMSA assays (Figure 4F and Supporting Information Figure S5D‐E). Furthermore, we certified TCF4 upregulation and the positive expression correlation between circ_SMAD4 and TCF4 in GC tissues (Supporting Information Figure S5F‐G). Thereafter, we downregulated TCF4 and observed declined CTNNB1 level and lowered protein levels of β‐catenin, CCND1 and c‐myc (Figure 4G‐I and Supporting Information S5H). Taken together, circ_SMAD4 interacted with TCF4 to affect CTNNB1 expression and WNT/β‐catenin activation.

### 3.5 Circ_SMAD4 recruited TCF4 to potentiate CTNNB1 transcription

**FIGURE 5:**
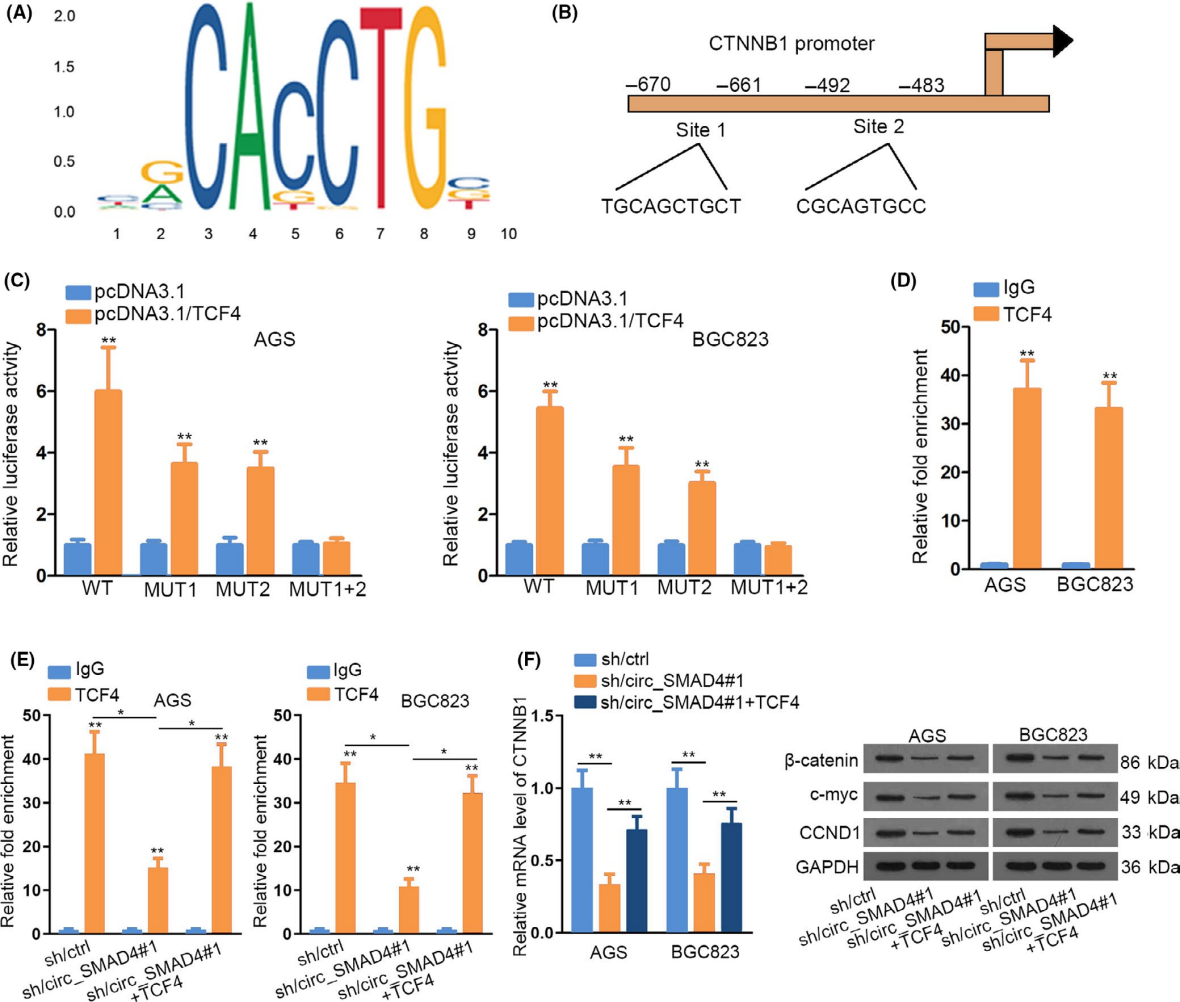
Circ_SMAD4 recruited TCF4 to potentiate CTNNB1 transcription. A, TCF4 motif obtained from JASPAR. B, JASPAR predicted two binding sites in CTNNB1 promoter for TCF4. C, Luciferase reporter assay determined the effect of TCF4 overexpression on the reporters containing different CTNNB1 promoter sequences. D, ChIP assay tested the interaction between TCF4 and CTNNB1 promoter. E, ChIP analysed the impact of circ_SMAD4/TCF4 on the binding of TCF4 to CTNNB1 promoter. F, qRT‐PCR and Western blot depicted the impact of TCF4 overexpression on circ_SMAD4 silence‐inhibited CTNNB1 mRNA and c‐myc, CCND1 and β‐catenin levels. *P < 0.05, **P < 0.01

Subsequently, we intended to confirm the impact of circ_SMAD4‐TCF4 interaction on CTNNB1 expression. By utilizing UCSC (http://genome.ucsc.edu/) and JASPAR (http://jaspar.genereg.net/), we obtained TCF4 binding motif and also found two potential TCF4 binding sites in CTNNB1 promoter (Figure 5A‐B). Further, luciferase reporter assay data revealed that upregulating TCF4 enhanced the luciferase activity of wild‐type CTNNB1 promoter, while such enhancement was partly mitigated when mutating site 1 or site 2 alone but completely offset when both sites were mutated (Figure 5C). These data corroborated that both sites were responsible for the interaction between TCF4 and CTNNB1 promoter. Subsequently, ChIP assay confirmed the binding between TCF4 and CTNNB1 promoter (Figure 5D). Interestingly, such binding was hindered upon circ_SMAD4 depression, but totally recovered under further augmentation of TCF4 (Figure 5E). However, the suppression of silenced circ_SMAD4 on CTNNB1 expression and the levels of β‐catenin, CCND1 and c‐myc were only partially counteracted after overexpressing TCF4 (Figure 5F and Supporting Information Figure S6A). Functionally, loss of TCF4 retarded GC cell growth, while such influence was then antagonized byCTNNB1 overexpression or LiCl treatment (Supporting Information Figure S6B‐D). All data substantiated that circ_SMAD4 potentiated CTNNB1 transcription via the recruitment of TCF4.

### 3.6 circ_SMAD4 upregulated CTNNB1 as a miR‐1276 sponge

**FIGURE 6:**
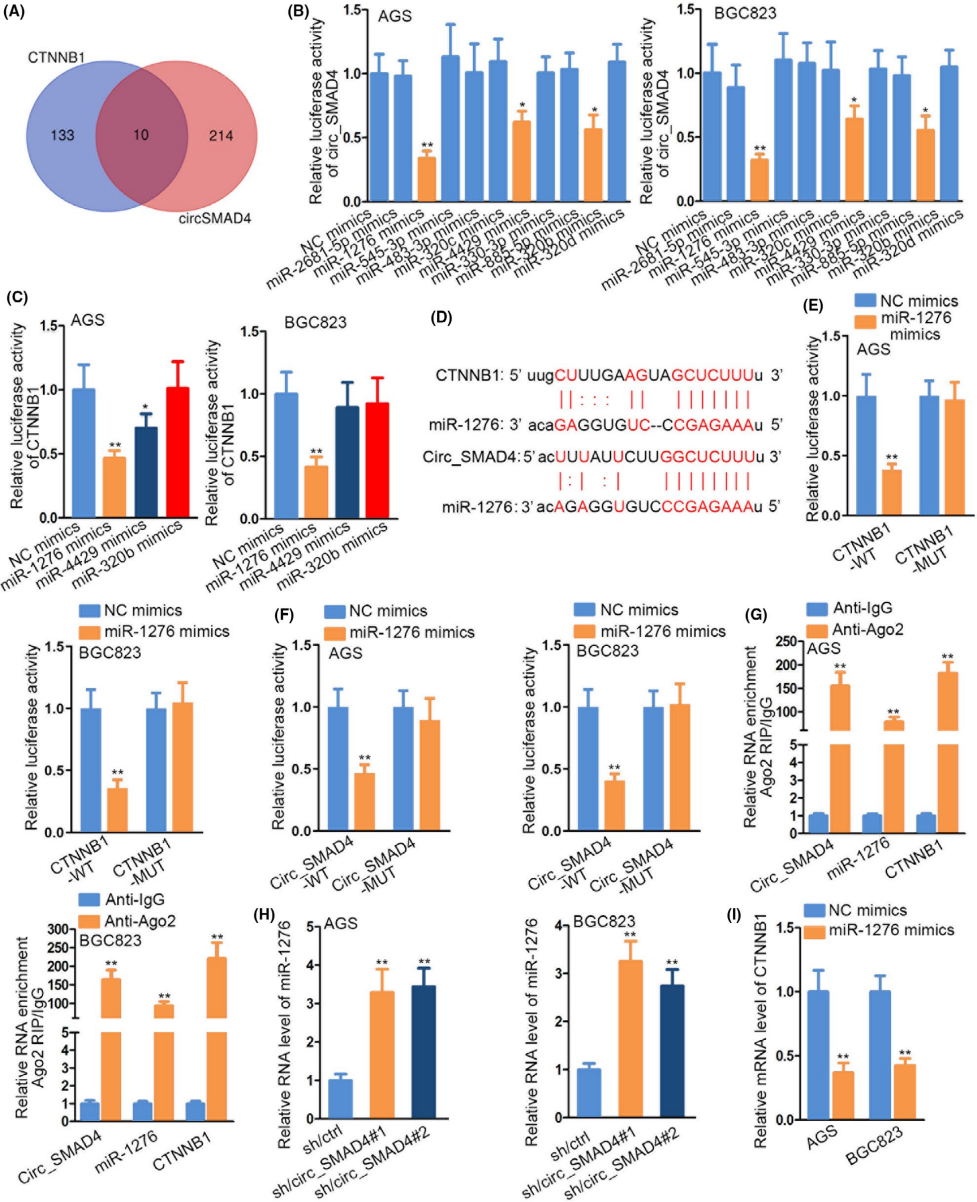
Circ_SMAD4 upregulated CTNNB1 by serving as a miR‐1276 sponge. A, With the predication of miRDB and starBase, potential miRNAs for circ_SMAD4 and CTNNB1 were unveiled. B, Luciferase reporter assay tested the luciferase activity of circ_SMAD4 under the respective overexpression of 10 miRNA mimics. C, Luciferase reporter assay examined the luciferase activity of CTNNB1 in the presence of 3 indicated miRNA mimics. D, MiR‐1276 binding sites in circ_SMAD4 and CTNNB1 were delineated. (E‐F) Luciferase reporter assay evaluated the luciferase activity of CTNNB1‐WT/MUT or circ_SMAD4‐WT/MUT upon miR‐1276 overexpression. G, Ago2‐RIP assay substantiated the existence of circ_SMAD4, miR‐1276 and CTNNB1 in Ago2 precipitates. H, qRT‐PCR investigated the influence of circ_SMAD4 inhibition on miR‐1276 level in AGS and BGC823 cells. I, qRT‐PCR measurement of CTNNB1 expression under miR‐1276 mimics. *P < 0.05, **P < 0.01

Since circ_SMAD4 modulated CTNNB1 expression not only depending on its nuclear function, we wondered whether cytoplasmic circ_SMAD4 also had some contribution to elevate CTNNB1 in GC cells. Further, considering the high potential for cytoplasmic circRNAs as a ceRNA, we focused on miRNAs that could bind to both circ_SMAD4 and CTNNB1. Through analysing starBase and miRDB, we discovered 10 miRNAs shared by circ_SMAD4 and CTNNB1 (Figure 6A), and three (miR‐1276, miR‐4429 and miR‐320b) were further selected since their overexpression decreased the luciferase activity of circ_SMAD4 (Figure 6B). Nevertheless, only miR‐1276 mimics could reduce the luciferase activity of CTNNB1 in both cells (Figure 6C). In addition, miR‐1276 expressed at a low level in GC tissues and cells (Supporting Information Figure S7A‐B), and its level was negatively related to circ_SMAD4 in GC samples (Supporting Information Figure S7C). Based on these data, we guessed miR‐1276 mediated the regulation of circ_SMAD4 on CTNNB1. Corresponding miR‐1276 binding sites in circ_SMAD4 and CTNNB1 were exhibited in Figure 6D. Luciferase reporter assay results displayed that only the luciferase activities of CTNNB1‐WT and circ_SMAD4‐WT declined upon miR‐1276 mimics (Figure 6E‐F). Additionally, miR‐1276 failed to affect the activity of SMAD4 3′UTR reporters (Supporting Information Figure S7D). Moreover, co‐existence of circ_SMAD4, miR‐1276 and CTNNB1 was found exclusively in the anti‐Ago2 group (Figure 6G). Furthermore, RT‐qPCR uncovered miR‐1276 upregulation under circ_SMAD4 repression and CTNNB1 downregulation under miR‐1276 elevation (Figure 6H‐I). As expected, inhibition of miR‐1276 cancelled the suppressive impact of circ_SMAD4 knock‐down on CTNNB1 mRNA, and β‐catenin, CCND1 and c‐myc proteins to certain extent (Supporting Information Figure S7E‐F). As shown in Supporting Information Figure S7G‐I, enhanced miR‐1276 led to proliferation inhibition and apoptosis stimulation in GC cells, while forced CTNNB1 expression or LiCl treatment counteracted above effects. So far, results indicated that circ_SMAD4 sequestered miR‐1276 to boost CTNNB1 expression in GC cells.

### 3.7 Circ_SMAD4 aggravated GC cell growth by augmenting CTNNB1 via miR‐1276 and TCF4

**FIGURE 7:**
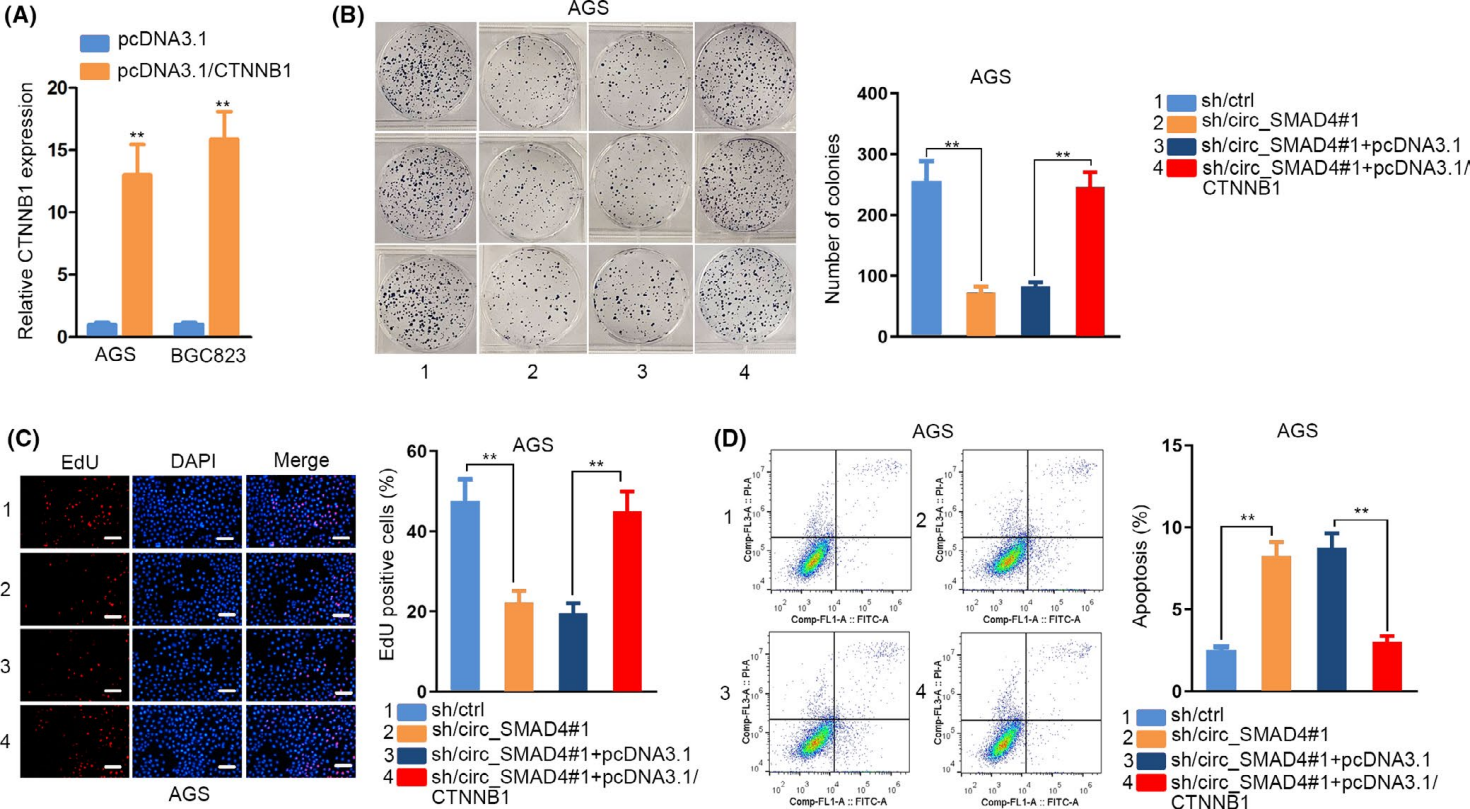
Augmentation of CTNNB1 restored GC cell growth inhibition induced by circ_SMAD4 suppression. A, qRT‐PCR confirmation of CTNNB1 overexpression in AGS and BGC823 cells. (B‐C) Colony formation and EdU assays tested cell proliferation under diverse conditions. D, Flow cytometry analysed the apoptosis of indicated cells. **P < 0.01

Previously, we disclosed that circ_SMAD4 boosted CTTNB1 expression in GC via both TCF4‐ and miR‐1276‐mediated manners. Here, we detected whether it functioned in GC also through these two pathways. It turned out that TCF4 upregulation or miR‐1276 inhibition alone resulted in partial recovery of circ_SMAD4 deficiency‐hampered cell growth, whereas the combined effect of them completely rescued the impeded cell growth induced by circ_SMAD4 interference (Supporting Information Figure S8A‐C). At length, we investigated whether circ_SMAD4 relied on CTNNB1 to function in GC cell growth. Before that, we validated that CTNNB1 was effectively upregulated by pcDNA3.1/CTNNB1 (Figure 7A). Consequently, CTNNB1 overexpression offsets the impacts of circ_SMAD4 depletion on cell proliferation and apoptosis (Figure 7B‐D). In sum, circ_SMAD4 drove GC tumorigenesis by upregulating CTNNB1 via miR‐1276 and TCF4.

## 4 DISCUSSION

In this study, circ_SMAD4 was selected as the most upregulated circRNA in GC through microarray assays. Further, we proved the overexpressed circ_SMAD4 in GC tissues and cells. With regard to its clinical features, we demonstrated that circ_SMAD4 contributed to shorter survival duration. Next, in vitro and in vivo assays functionally displayed that circ_SMAD4 exerted oncogenic effect on tumorigenesis in GC. These findings linked circ_SMAD4 to GC pathogenesis.

Mechanically, we unveiled WNT/β‐catenin pathway as the downstream of circ_SMAD4 in GC. Previously, the modulation of circRNAs on this pathway has been substantiated by various reports.^28^ The specific role of circRNAs varies according to their cellular distribution.^29^ Presently, we discovered circ_SMAD4 located in both nucleus and cytoplasm of GC cells. Subsequently, the nuclear circ_SMAD4 was uncovered to influence CTNNB1 transcription. Inspired by the previous hypothesis that circRNAs could interact with specific nuclear proteins to transcriptional regulate gene expressions.^22^ We searched the binding proteins of circ_SMAD4 and discovered TCF4. TCF4 can bind to β‐catenin to trigger WNT/β‐catenin pathway.^30^, ^31^ Interestingly, here we proved that nuclear circ_SMAD4 could interact with TCF4 and then recruit TCF4 to CTNNB1 promoter to potentiate CTNNB1 transcription, resulting in WNT/β‐catenin signalling transmission. Similar function of other circRNAs has also been described by a number of reports. For instance, circ‐DONSON initiated SOX4 transcription through enhancing the recruitment of NURF complex to SOX4 promoter.^22^ Also, circAnks1a recruited YBX1 to Vegfb promoter to activate Vegfb transcription.^23^ Additionally, rescue assay results verified that TCF4 contributed to GC cell growth by CTNNB1‐activated WNT/β‐catenin pathway. However, we uncovered that TCF4‐induced elevation of CTNNB1 could not offset the suppression of silenced circ_SMAD4 on CTNNB1, indicating circ_SMAD4 regulated CTNNB1 through another pathway.

It is proposed that cytoplasmic circRNAs, mRNAs and lnRNAs share and compete for the binding of miRNAs to establish a competing endogenous network which post‐transcriptionally modulates protein‐coding genes.^32^, ^33^ For example, cytoplasmic circAKT3 promoted PIK3R1 by sponging miR‐198 in GC cells.^34^ Current study manifested a post‐transcriptional modulation of circ_SMAD4 on CTNNB1 as a sponge of miR‐1276. Further, we proved that circ_SMAD4 affected WNT/β‐catenin pathway by miR‐1276/CTNNB1 axis. Moreover, miR‐1276 inhibition and TCF4 upregulation completely offset the impacts of interfered circ_SMAD4 on CTNNB1 expression and GC cell growth.

In conclusion, our work first illuminated the pro‐growth role of circ_SMAD4 in GC through activating CTNNB1‐dependent WNT/β‐catenin signalling via interacting with miR‐1276 and TCF4. Consequently, targeting circ_SMAD4 might be a promising method for treating GC. However, how WNT/β‐catenin pathway affects circ_SMAD4 expression in turn still remains covered, which is the biggest limitation of our present work.

## CONFLICT OF INTEREST

The authors addressed no competing interests.

## AUTHOR CONTRIBUTIONS

Liyan Wang designed this study and analysed the data. Bin Li, Xiaoyuan Yi, Xuhua Xiao, Qinghua Zheng and Lei Ma were responsible for the experiment and figures. All authors contributed to write and review the manuscript.

## Supporting information

Figures S1–S8

## Data Availability

Research data are all within the present manuscript and the additional files.
