# Supplementary material for: Circ_SMAD4 promotes gastric carcinogenesis by activating wnt/β‐catenin pathway
Source: Cell Prolif. 2021 Jan 17;54(3):e12981. doi: 10.1111/cpr.12981 (PMC7941240; doi:10.1111/cpr.12981)
Supplement: Supplementary file 1 — Figures S1–S8 [file CPR-54-e12981-s001.docx]

**Figure S1**

(A) Sanger sequencing of the splice junction of circ_SMAD4. (B-C) AGE analysis of circ_SMAD4 or GAPDH in different products. (D) SMAD4 expression in 40 GC tissues and paired non-cancerous tissues was determined by qRT-PCR. **P < 0.01.

**
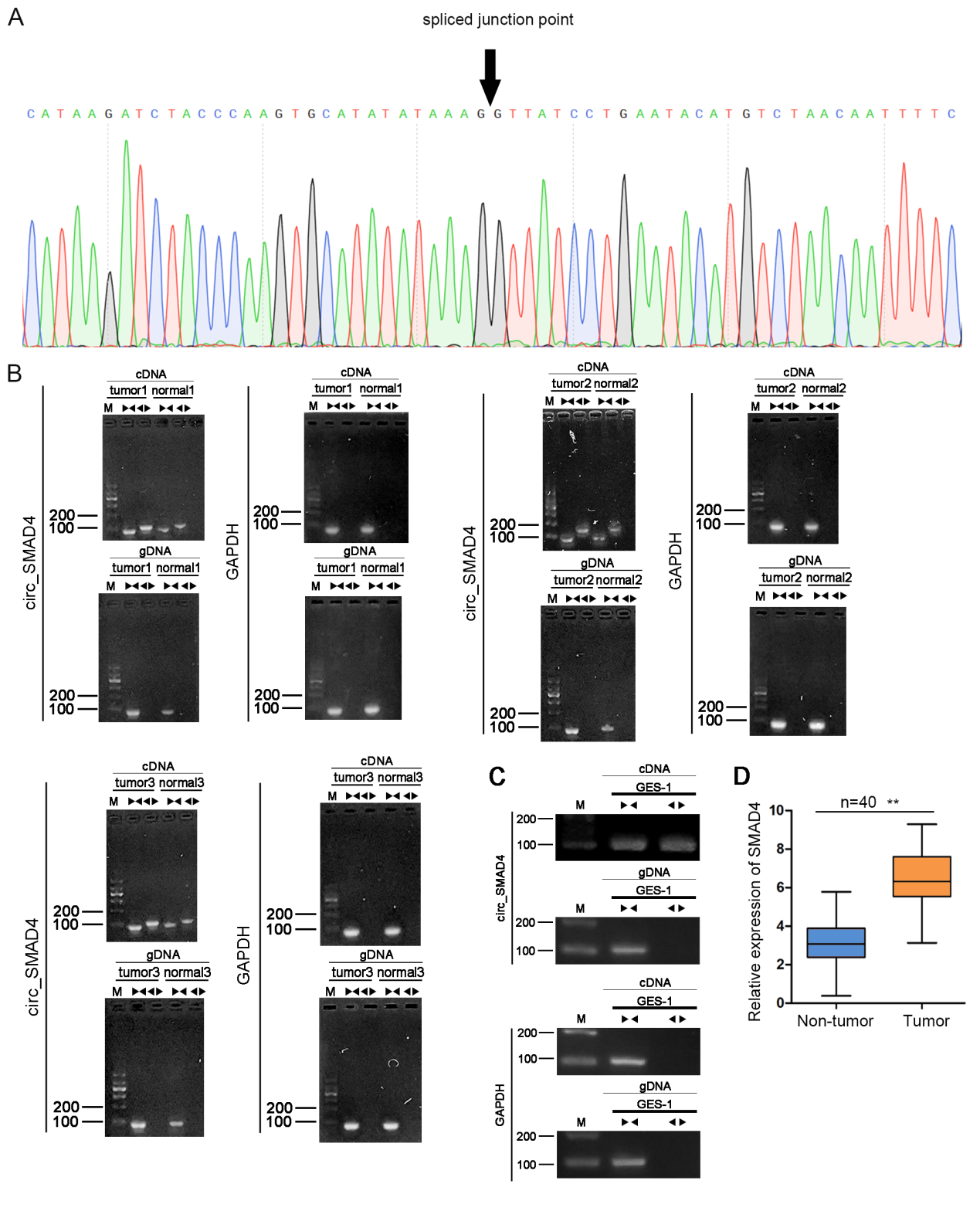
**

**Figure S2**

(A) Western blots showed that SMAD4 protein level was not affected by circ_SMAD4 knockdown. (B) The efficiency of circ_SMAD4 overexpression was detected via RT-qPCR. (C-D) Colony formation and EdU assays validated the influence of circ_SMAD4 overexpression on GC cell proliferation. **P < 0.01.

**
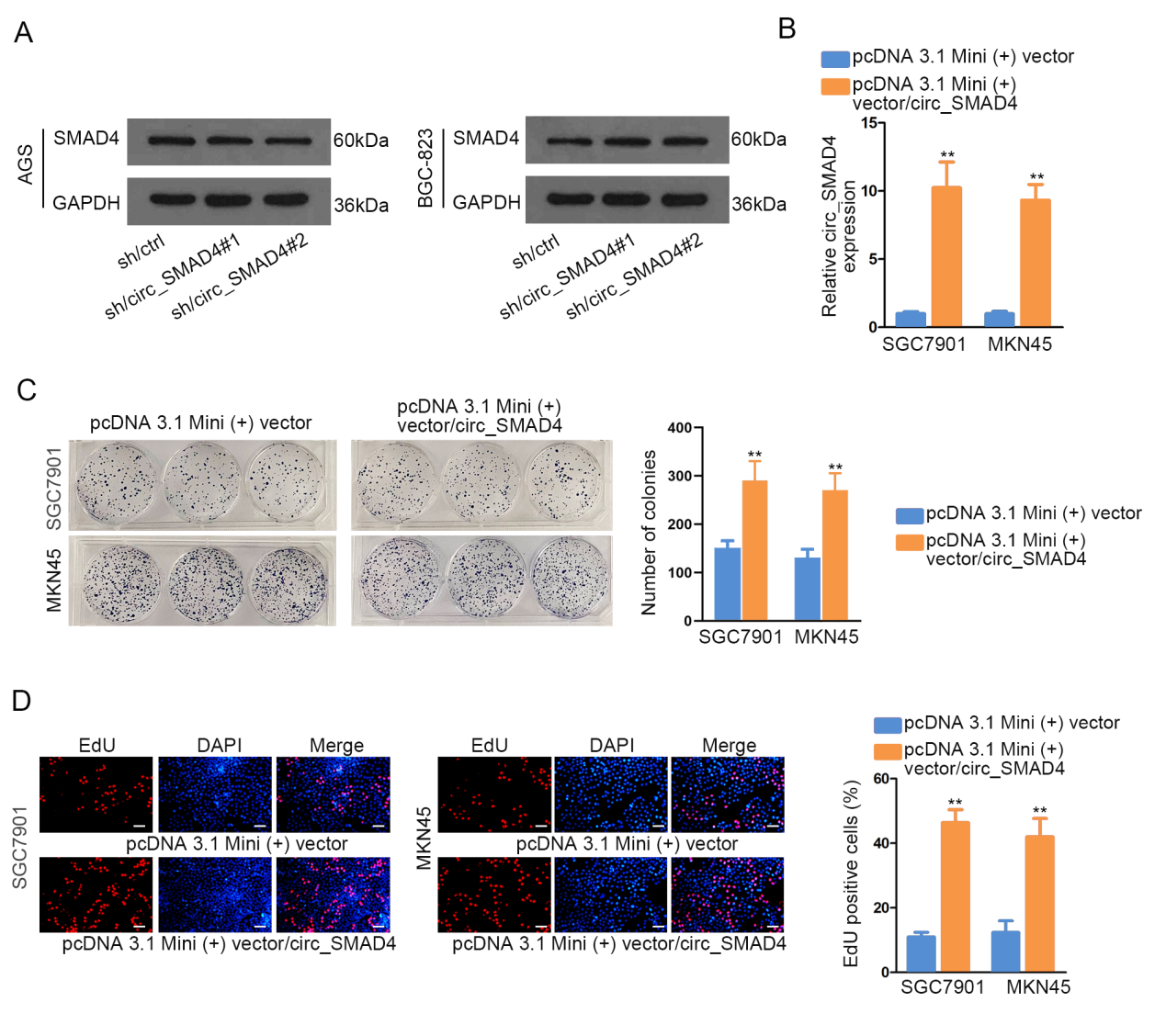
**

**Figure S3**

(A-C) After treatment with XAV-939 in AGS and BGC823 cells, cell proliferation and apoptosis were detected by colony formation, EdU and flow cytometry assays. **P < 0.01.

**
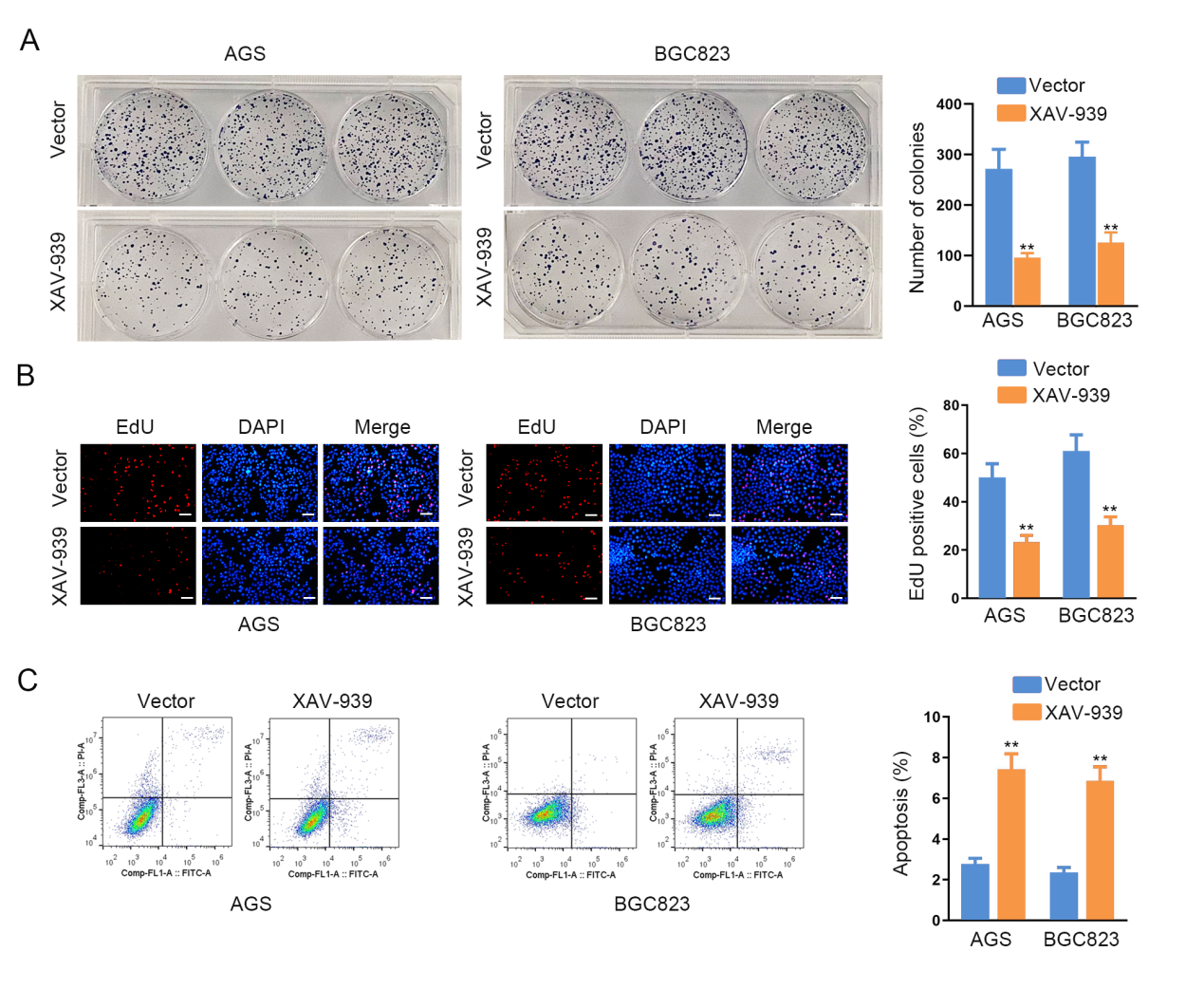
**

**Figure S4**

(A-C) Colony formation, EdU and flow cytometry experiments tested cell proliferation and apoptosis under indicated contexts. **P < 0.01.

**
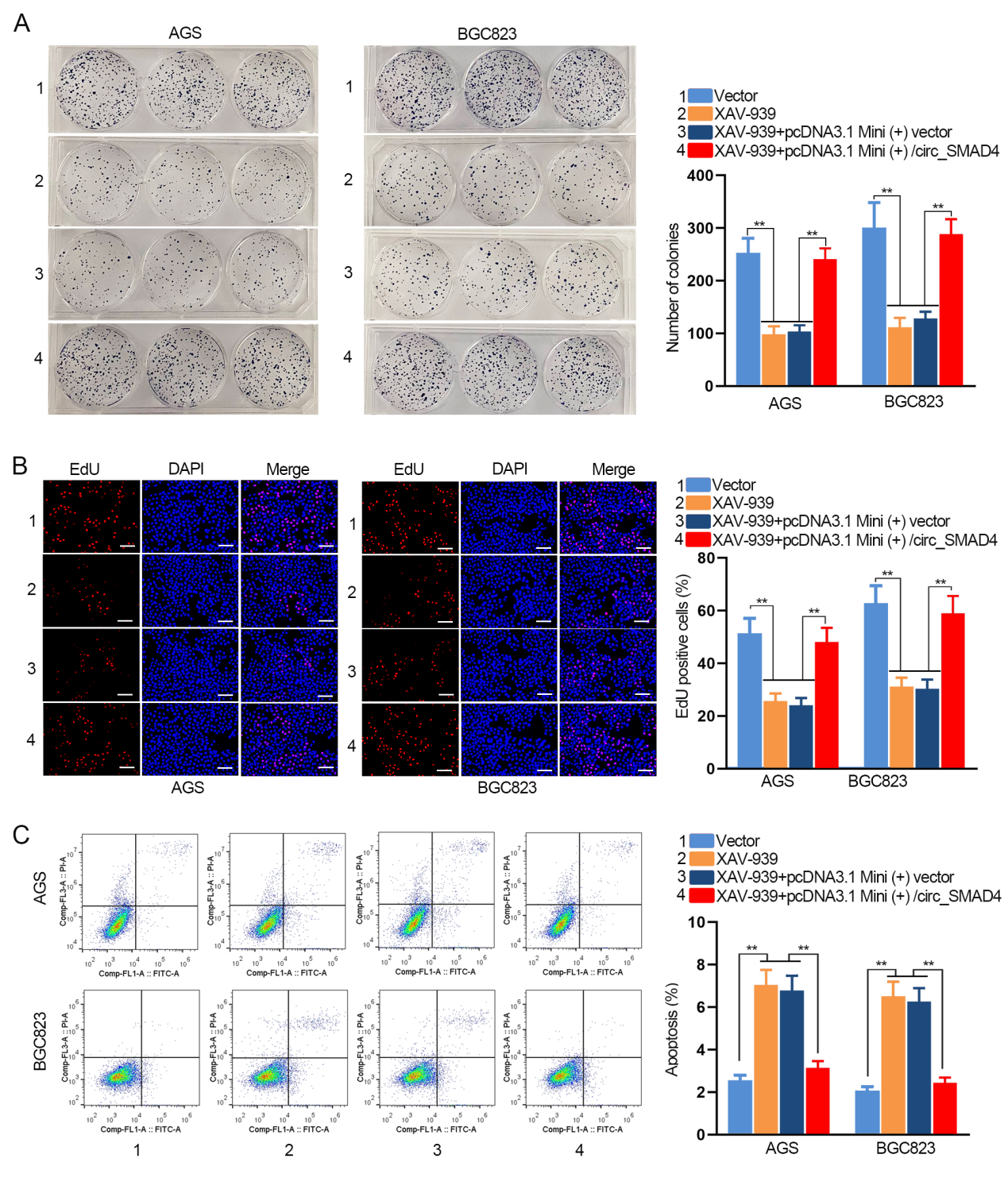
**

**Figure S5**

(A) The expression of CTNNB1 in 40 GC tissues and matched non-tumor tissues was detected via RT-qPCR. (B) The correlation of CTNNB1 with circ_SMAD4 in 40 GC samples was analyzed through Pearson’s correlation analysis. (C) The quantification of the protein levels of β-catenin, c-myc and CCND1 under circ_SMAD4 inhibition. (D-E) RNA pull-down and EMSA assays verified the interaction of TCF4 with circ_SMAD4. (F) The expression of TCF4 in GC tissues and matched non-tumor tissues was detected via RT-qPCR. (G) The correlation of TCF4 with circ_SMAD4 in 40 GC samples was analyzed through Pearson’s correlation analysis. (H) The quantification of the protein levels of β-catenin, c-myc and CCND1 under TCF4 suppression. **P < 0.01.

**
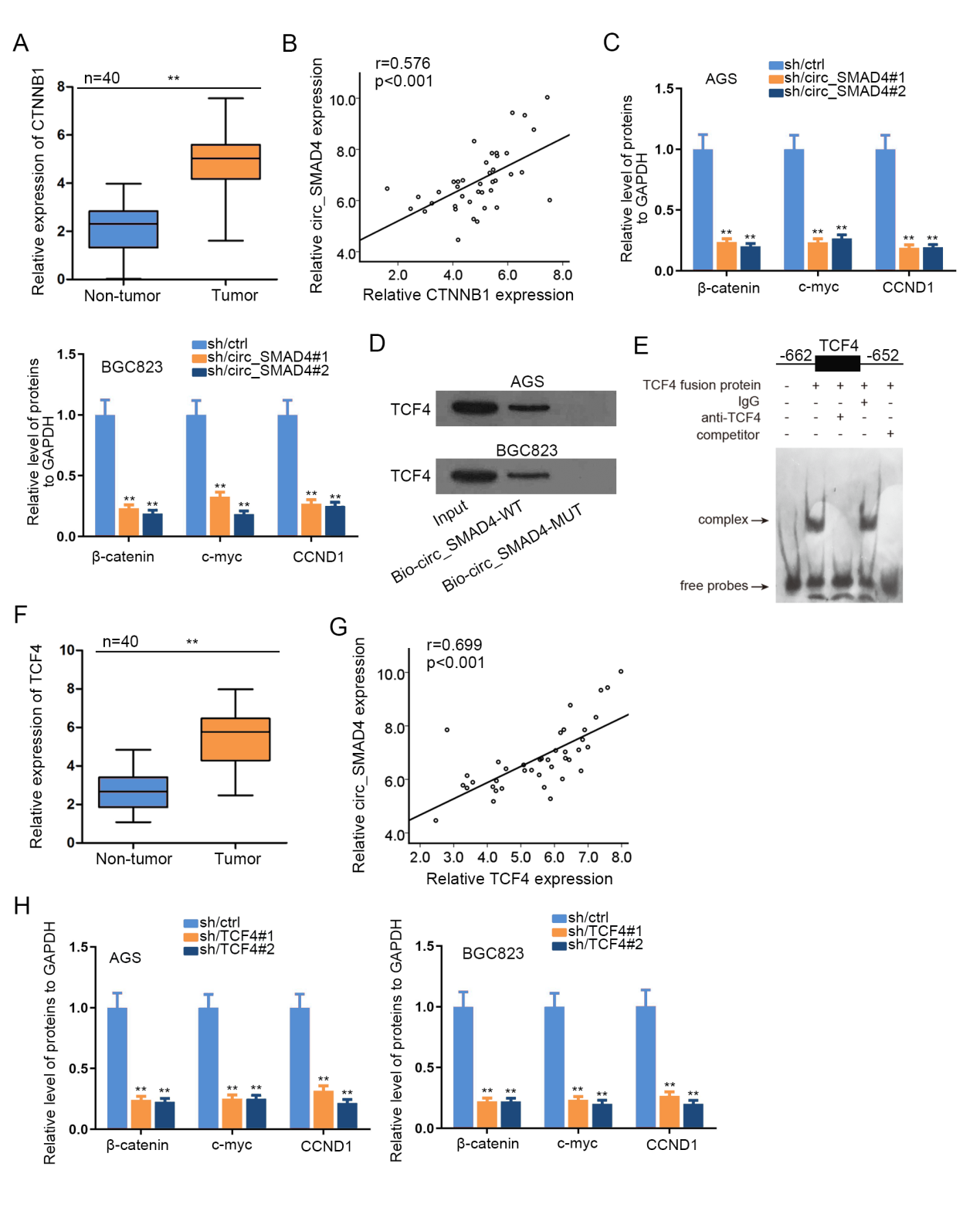
**

**Figure S6**

(A) The quantification of the protein levels of β-catenin, c-myc and CCND1 under circ_SMAD4 interference or and TCF4 overexpression. (B-D) Colony formation, EdU and flow cytometry experiments examined cell growth under different contexts. **P < 0.01.

**
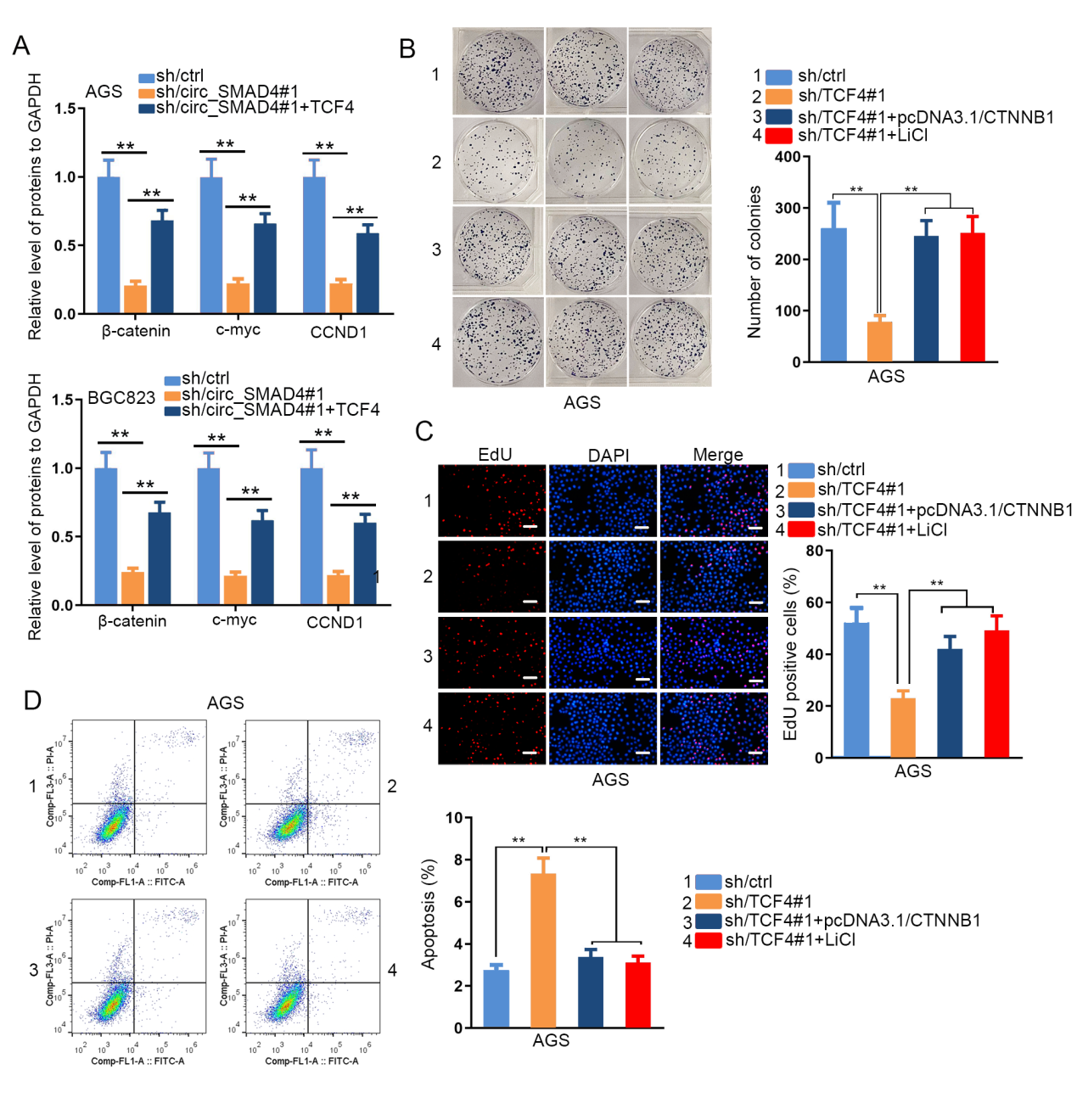
**

**Figure S7**

(A-B) The expression of miR-1276 in 40 pairs of clinical samples and GC cells was detected via RT-qPCR. (C) The correlation of miR-1276 with circ_SMAD4 in 40 GC tumor tissues was analyzed through Pearson’s correlation analysis. (D) Luciferase reporter assay confirmed the effect of miR-1276 upregulation on SMAD4 3’UTR reporter. (E-F) qRT-PCR and western blot validated that miR-1276 inhibition recovered the suppression of circ_SMAD4 depression on CTNNB1, c-myc, CCND1 and β-catenin. (G-I) Colony formation assay, EdU assay and flow cytometry analysis displayed that the impact of miR-1276 inhibition on GC cell proliferation or apoptosis was offset following pcDNA3.1/CTNNB1 or LiCl treatment. *P < 0.05, **P < 0.01. n.s.: no significance.

**
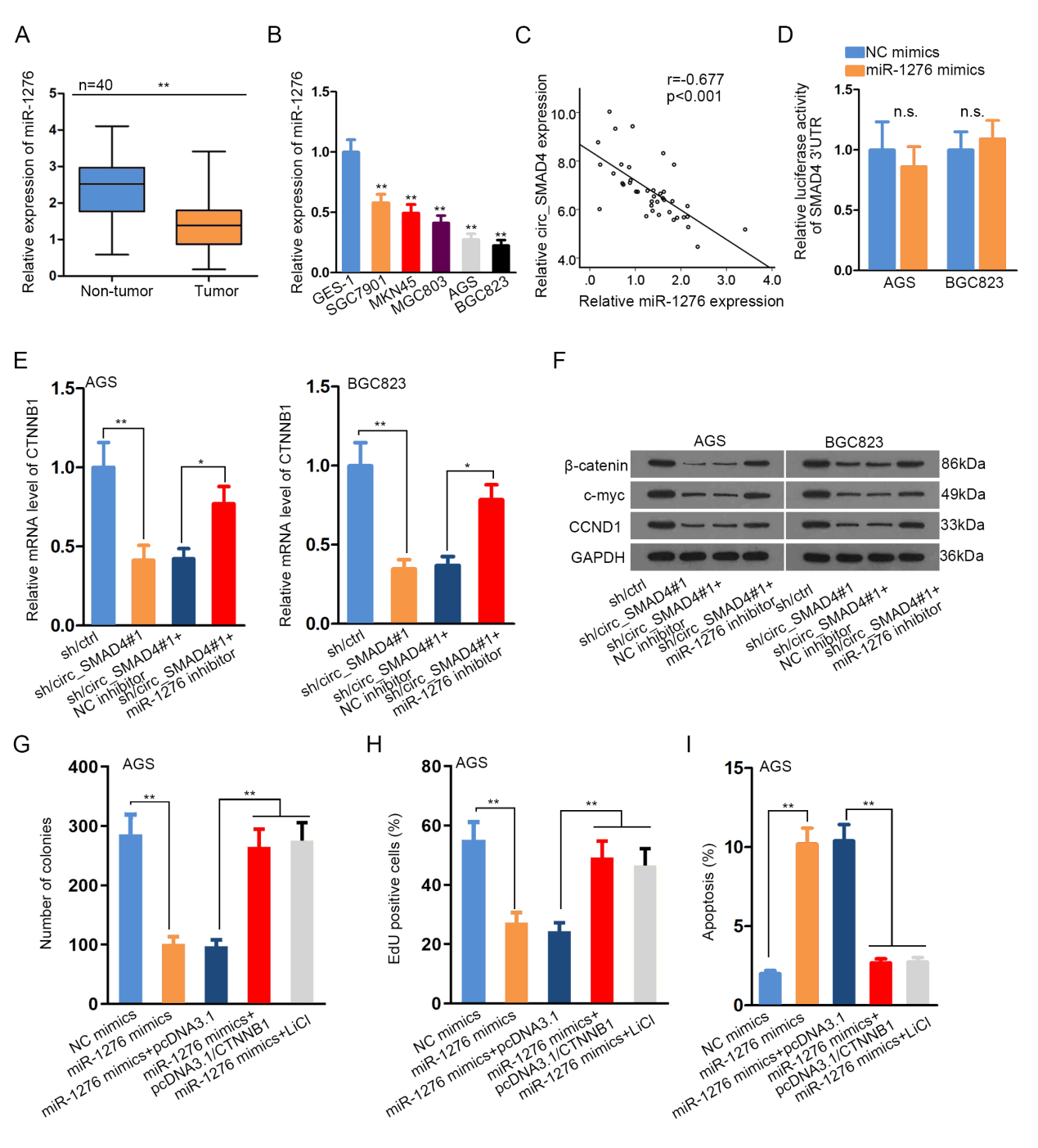
**

**Figure S8**

(A-C) The influences of TCF4 upregulation or/and miR-1276 inhibition on circ_SMAD4 depletion-affected GC cell proliferation and apoptosis were tested by colony formation assay, EdU assay and flow cytometry analysis. *P < 0.05, **P < 0.01.

**
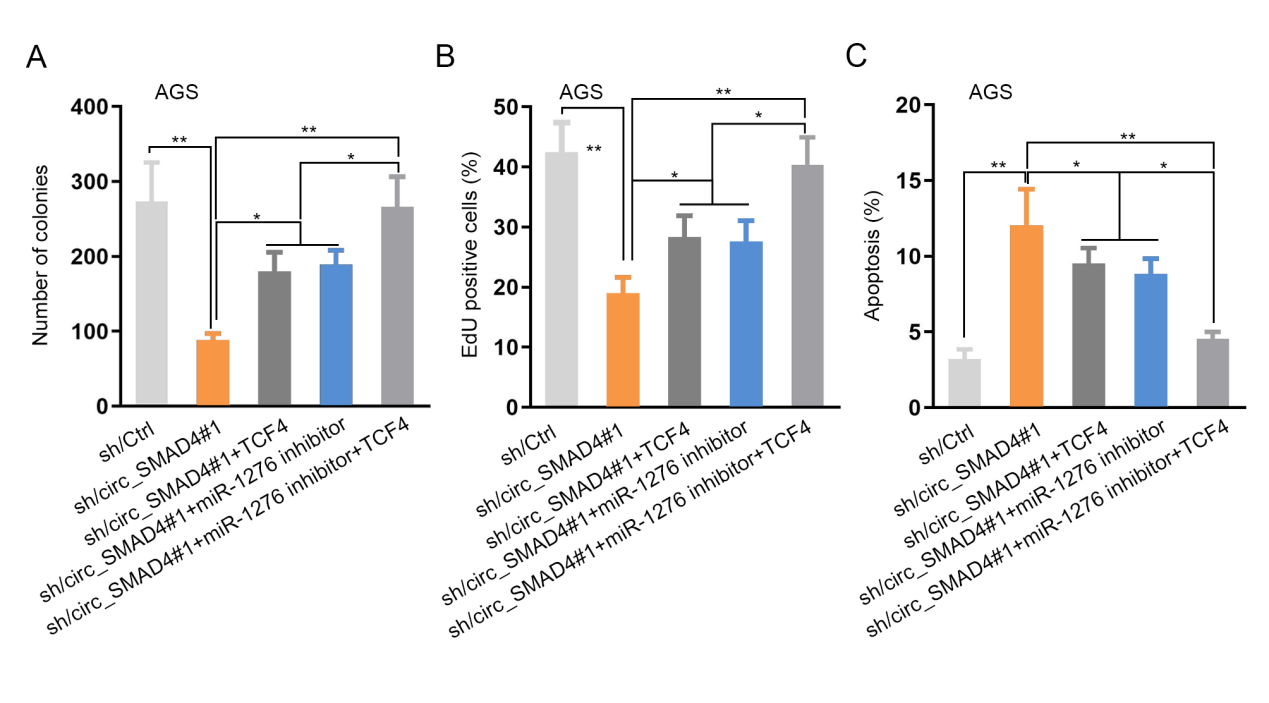
**
